# Unravelling the Microbiome of Eggs of the Endangered Sea Turtle *Eretmochelys imbricata* Identifies Bacteria with Activity against the Emerging Pathogen *Fusarium falciforme*

**DOI:** 10.1371/journal.pone.0095206

**Published:** 2014-04-17

**Authors:** Jullie M. Sarmiento-Ramírez, Menno van der Voort, Jos M. Raaijmakers, Javier Diéguez-Uribeondo

**Affiliations:** 1 Departamento de Micología, Real Jardín Botánico-CSIC, Madrid, Spain; 2 Laboratory of Phytopathology, Wageningen University, Wageningen, The Netherlands; 3 Department of Microbial Ecology, Netherlands Institute of Ecology (NIOO-KNAW), Wageningen, The Netherlands; University of Illinois, United States of America

## Abstract

Habitat bioaugmentation and introduction of protective microbiota have been proposed as potential conservation strategies to rescue endangered mammals and amphibians from emerging diseases. For both strategies, insight into the microbiomes of the endangered species and their habitats is essential. Here, we sampled nests of the endangered sea turtle species *Eretmochelys imbricata* that were infected with the fungal pathogen *Fusarium falciforme*. Metagenomic analysis of the bacterial communities associated with the shells of the sea turtle eggs revealed approximately 16,664 operational taxonomic units, with Proteobacteria, Actinobacteria, Firmicutes and Bacteroidetes as the most dominant phyla. Subsequent isolation of Actinobacteria from the eggshells led to the identification of several genera (*Streptomyces*, *Amycolaptosis*, *Micromomospora Plantactinospora* and *Solwaraspora*) that inhibit hyphal growth of the pathogen *F. falciforme*. These bacterial genera constitute a first set of microbial indicators to evaluate the potential role of microbiota in conservation of endangered sea turtle species.

## Introduction

Sea turtles are one of the most endangered groups of animals worldwide with only seven species left [1]. Incidental by-catch, disturbance of nesting beaches, pollution and diseases are major causes of drastic population declines [2]. Among the emerging diseases, the fungal pathogens *Fusarium falciforme* and *F. keratoplasticum* are an increasing threat to sea turtle nests, especially to those experiencing environmental stress [3].

Several conservation strategies have been proposed to mitigate the impact of pathogens on endangered species. For example, establishment of *ex situ* colonies and ‘habitat bioaugmentation and biotherapy’ have been proposed to prevent dispersal of the fungal pathogen *Batrachotrichum dendrobatidis* in amphibian populations [4]. The latter two strategies encompass the use of protective microbiota, either indigenous or introduced, to limit pathogen infection and spread. These two approaches are adopted in agriculture to control plant diseases [5]–[8]. Also in mammals, the role of gut microbiota in health and disease is now widely studied [9]–[13]. In nature conservation programs, however, these approaches are not common yet. This is due, in part, to a lack of knowledge of the overall diversity of microbiota associated with endangered species and their role, if any, in protecting their hosts against pathogen infection [14].

The structure of microbial communities of different hosts, their genetic diversity, and ecological roles have been studied combining culture-based analysis with polymerase chain reaction (PCR) techniques [15]–[17]. For example, the high-density 16S ribosomal DNA (rDNA) oligonucleotide microarray, referred to as the PhyloChip [18], [19] combined with bacterial isolations has helped identifying key bacterial and archaeal community members in the rhizosphere of plants grown in disease-suppressive soils [20]. In sea turtles, a limited number of culture-based and biochemical studies have allowed describing taxa of bacteria associated with egg failure in several species [21]–[27]. These studies have listed and reported on potentially pathogenic bacteria from unhatched sea turtle eggs. However, full characterization of the microbial community and its effect on hatching of sea turtle eggs has, to our knowledge, never been conducted.

In this study, we investigated the microbial community associated with *Fusarium*-infected eggs of the critically endangered sea turtle species *Eretmochelys imbricate*. To that end, we collected eggs from the nesting beach La Playita at Machalilla National Park, Ecuador, in order to survey for bacteria with antifungal activity. For this purpose, PhyloChip analysis was used to identify the bacterial community associated with the turtle eggs. Based on these analyses, targeted isolations of specific bacterial genera were conducted using culture-based techniques followed by *in vitro* assays to determine the potential antagonistic activity of the selected indigenous microbiota against *F. falciforme*, the fungal pathogen of sea turtle eggs [3].

## Results

### Fungal isolation and molecular characterization

**Figure 1:**
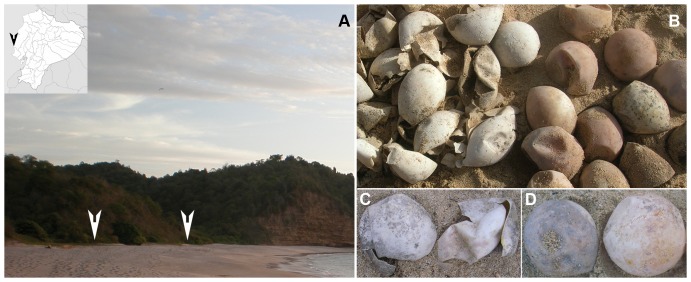
Sea turtle nesting area sampled for this study. A) Nests of the sea turtle *Eretmochelys imbricata* in La Playita beach at Machalilla National Park, Ecuador. B) Nest containing hatched and unhatched *Fusarium*-infected eggs. C) *Fusarium*-infected hatched eggs. D) *Fusarium*-infected unhatched eggs.

A total of 10 fungal isolates were obtained from the eggshells (Figure 1, S1) and initially identified as *F. solani* based on NCBI BLAST analysis of the ITS nrDNA sequences (Table 1). Phylogenetic analysis of the ITS nrDNA showed that the 10 fungal isolates clustered within the previously described species *F. falciforme* (Table 1 and Figure S2).

**Table 1:** *Fusarium falciforme* isolates from eggshells of the sea turtle species *Eretmochelys imbricata*.

Strain	Source	aGenBank Accession	bMaximum identity
326FUS	Hatched egg	KF179246	100%
327FUS	Hatched egg	KF179247	99%
328FUS	Hatched egg	KF179248	100%
329FUS	Hatched egg	KF179249	100%
330FUS	Hatched egg	KF179250	100%
331FUS	Hatched egg	KF179251	99%
332FUS	Unhatched egg	KF179252	100%
333FUS	Unhatched egg	KF179253	100%
334FUS	Unhatched egg	KF179254	100%
335FUS	Unhatched egg	KF179255	100%

a GenBank accession number of the *F. falciforme* isolates.

b BLAST hit corresponds to the NCBI nucleotide database. All the blast hits corresponded with *F. solani* strains.

### Bacterial isolation and DNA extraction from sea turtle egg shells

The number of culturable aerobic bacteria, enumerated on 1/10^th^ strength Tryptic Soy Agar (TSA) medium, ranged from 3.1×10^7^ to 8.7×10^7^ Colony Forming Units per area of eggshell (CFU/cm^2^) from hatched and unhatched turtle eggs respectively. The population density of culturable Actinobacteria, enumerated on semi-selective medium glycerol-arginine agar (GA), ranged from 1.2×10^4^ to 2×10^5^ CFU/cm^2^ from hatched and unhatched eggs, respectively (Table S1). The Actinobacteria comprised on average, 0.2% of the total aerobic bacteria enumerated on 1/10^th^ TSA (Table S1).

### PhyloChip analysis

**Figure 2:**
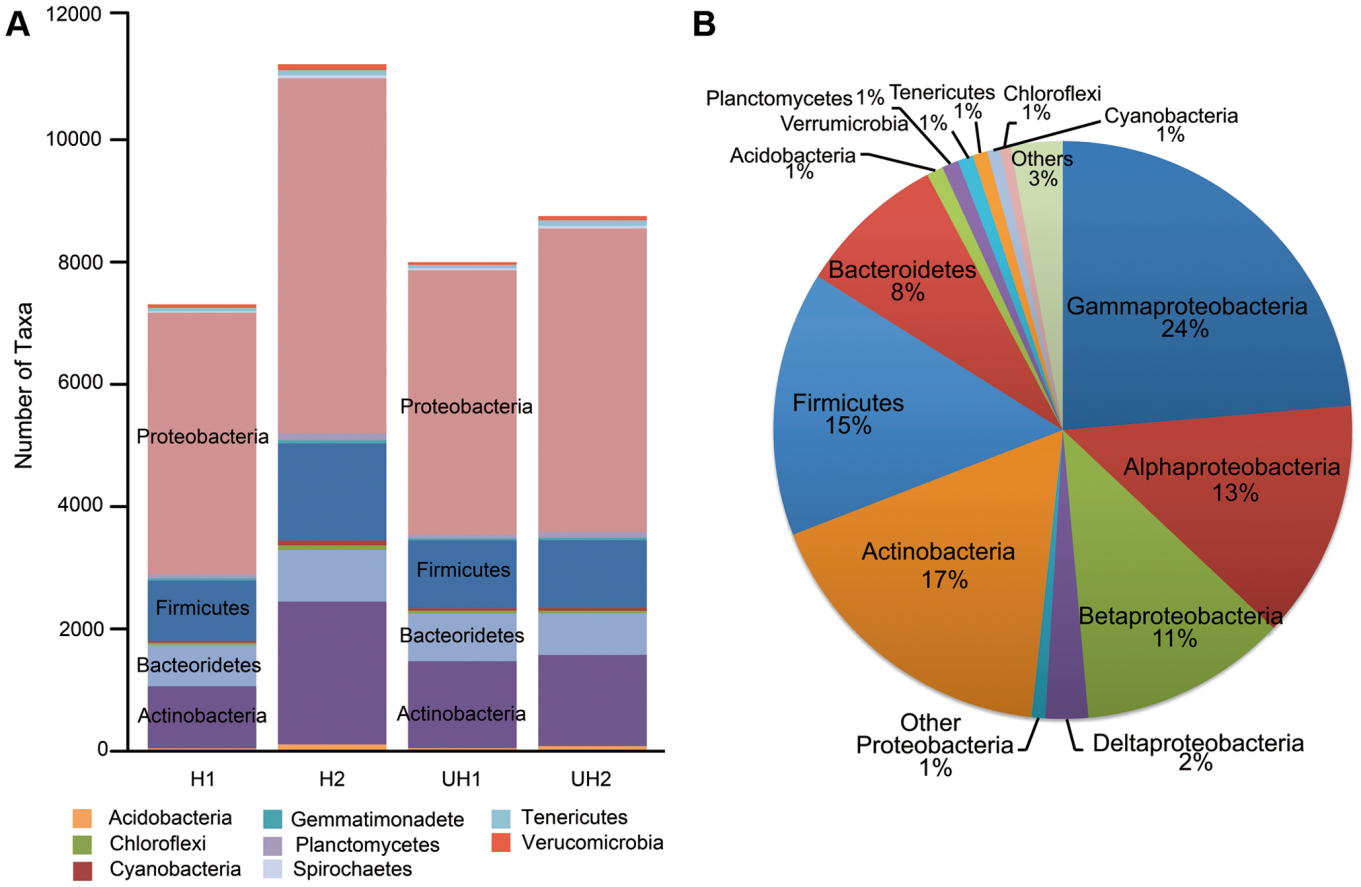
Composition of the microbial community of shells of *Fusarium*-infected eggs detected by the PhyloChip analysis. A) Number of OTUs per phylum detected on hatched (H) and unhatched (UH) eggshells collected from two nests (numbers 1 and 2). Values with >0.25% of occurrence. B) Average distribution of OTUs for all the samples (*n* = 4).

PhyloChip-based metagenomic analysis of the bacterial communities associated with the eggshells revealed the presence of 16,664 operational taxonomic units (OTUs). On average, Proteobacteria (52%), Actinobacteria (17%), Firmicutes (15%) and Bacteroidetes (8%) were detected as the most dominant phyla (Figure 2A, B). No significant differences were detected in overall bacterial phyla composition between hatched and unhatched eggs or between the two nests (Figure 2A). At family level, however, significant (Welsh test, p<0.01; *r* = 0.75, Anosim) differences in abundance between the two nests were found for the Pseudomonadaceae, which comprised 24% of the Gammaproteobacteria (Figure S3A; Table S2). Furthermore, the Flavobacteriaceae, which comprised 51% of the Bacteroidetes detected, were significantly (*r* = 1, Anosim) more abundant on shells of hatched eggs than those of unhatched eggs (Figure S3B). Within the Flavobacteriaceae, *Chryseobacterium* was the second most abundant genus (10%) and *C. indologenes* and *C. gleum* were the most represented species (Welch test, p<0.01) in our PhyloChip analysis (Table S3). These two species represented 7 and 5% of the genus *Chryseobacterium*, respectively.

### *In vitro* activity assay and BOX-PCR based identification of Actinobacteria

The Actinobacteria was the second most abundant bacterial phylum detected on the sea turtle eggshells. Given their well-documented ability to produce an array of antibacterial and antifungal compounds [33]-[35], we isolated Actinobacteria from the eggshells and determined their activity against *F. falciforme*. Out of a total of 98 randomly selected Actinobacteria isolates from hatched (*n* = 69) and unhatched eggs (*n* = 29), thirty-one inhibited hyphal growth of *F. falciforme* (isolate 331FUS). Among these 31 isolates with antifungal properties, 23 different haplotypes were identified by BOX-PCR fingerprinting. Subsequent 16S rDNA sequencing and phylogenetic analysis indicated that these isolates belong to the genera *Streptomyces* (16), *Amycolaptosis* (3), *Micromonospora* (1), *Plantactinospora* (4) and *Solwaraspora* (5) (Table 2). A total of 25 out of 31 of the antagonistic Actinobacteria isolates were obtained from hatched eggs. Out of the 6 isolates obtained from unhatched eggs, 1 corresponded to the genus *Planctactinospora* and the other five to *Streptomyces*.

**Table 2:** 16S rDNA sequence identities of the Actinobacteria, isolated from sea turtle eggshells, that inhibited the hyphal growth of *Fusarium falciforme*.

Isolate ACT^a^	Source	Identity of best BLAST hit^b^	GenBank accesion	Score	Identity
2	Hatched egg	*Micromonospora* sp.	KF179216	979	99%
121	Hatched egg	*Micromonospora* sp.	KF179221	1246	99%
13	Hatched egg	*Plantactinospora* sp.	KF179222	1222	98%
14	Hatched egg	*Plantactinospora* sp.	KF179223	1222	98%
20	Hatched egg	*Plantactinospora* sp.	KF179224	1226	98%
125	Unhatched egg	*Plantactinospora* sp.	KF179225	1232	99%
1	Hatched egg	*Solwaraspora* sp.	KF179226	1260	100%
16	Hatched egg	*Solwaraspora* sp.	KF179227	1260	100%
19	Hatched egg	*Solwaraspora* sp.	KF179228	1260	100%
23	Hatched egg	*Solwaraspora* sp.	KF179229	1245	100%
108	Hatched egg	*Solwaraspora* sp.	KF179230	1245	100%
145	Hatched egg	*Amylocolaptosis coloradensis*	KF179218	1134	99%
151	Hatched egg	*Amylocolaptosis coloradensis*	KF179219	1238	99%
152	Hatched egg	*Amylocolaptosis coloradensis*	KF179220	1245	99%
147	Hatched egg	*Streptomyces mutabilis*	KF179231	1265	100%
150	Hatched egg	*Streptomyces albogriseolus.*	KF179232	1250	99%
146	Hatched egg	*Streptomyces variabilis*	KF179217	1065	100%
148	Hatched egg	*Streptomyces variabilis.*	KF179233	1215	100%
149	Hatched egg	*Streptomyces variabilis*	KF179234	1264	100%
153	Hatched egg	*Streptomyces variabilis*	KF179235	1273	100%
154	Hatched egg	*Streptomyces variabilis*	KF179236	1270	100%
155	Hatched egg	*Streptomyces variabilis*	KF179237	1273	100%
156	Hatched egg	*Streptomyces variabilis*	KF179238	1273	100%
157	Hatched egg	*Streptomyces variabilis*	KF179239	1273	100%
162	Hatched egg	*Streptomyces variabilis*	KF179240	1278	100%
164	Unhatched egg	*Streptomyces variabilis*	KF179241	1272	100%
166	Unhatched egg	*Streptomyces variabilis*	KF179242	1269	100%
167	Unhatched egg	*Streptomyces variabilis*	KF179243	1281	100%
169	Unhatched egg	*Streptomyces variabilis*	KF179244	1244	100%
170	Unhatched egg	*Streptomyces variabilis*	KF179245	1277	100%

a ACT corresponds to the acronym of the Actinobacterial isolates.

b BLAST hit corresponds to the Greengenes database (greengenes.lbl.gov/cgi-bin/nph-blast_interface.cgi).

The data represent the best BLAST hit with 16S rDNA sequences from the GreenGenes database (greengenes.lbl.gov/cgi-bin/nph-blast_interface.cgi).

Based on phylogenetic analysis of the 16S sequences, the antagonistic *Streptomyces* isolates clustered in three different groups within the 364 *Streptomyces* OTUs detected by the PhyloChip (Figure S4). The antagonistic isolates classified as *S. mutabilis* and *S. albogriseolus* clustered with OTUs classified as the same species detected by the PhyloChip (BS = 74% and BS<50% respectively). The antagonistic *S. variabilis* isolate clustered with OTUs detected by the PhyloChip classified as *S. variabilis* and *S. aureofaciens* (BS = 58%).

Similarly, phylogenetic analysis of the 16S sequences of the antagonistic isolates belonging to *Amycolaptosis* sp. and Micromonosporaceae, *i.e*., *Micromonospora* sp., *Plantactinospora* sp. and *Solwaraspora* sp., could be linked with representatives of each of these four Actinobacterial genera detected by PhyloChip analysis (Figure S5A, S5B), The antagonistic isolates identified as *Micromonospora* sp. clustered with seven OTUs of different species (BS<50%), and those identified as *Solwaraspora* sp. grouped with one OTU of this genus detected by the PhyloChip (BS<50%). The antagonistic isolate identified as *Plantactinospora* sp. clustered with one OTU of the species *Plantactinospora mayteni* (BS = 67%) and the *Amycolaptosis coloradensis* isolates clustered with representatives of this species detected by the PhyloChip (BS<50%).

## Discussion

In this study, we described the microbial community of *Fusarium*-infected sea turtle eggs from the critically endangered species *Eretmochelys imbricata*. Due to the extreme difficulties to obtain samples and export permits from authorities for studies on endangered and critically endangered species, only four eggs were allowed to be collected. Hence, the results presented here provide a first ‘glimpse’ into the microflora associated with sea turtle eggs.

The PhyloChip analyses showed that the bacterial community associated with the eggs is mainly represented by the phyla Proteobacteria, Actinobacteria, Firmicutes and Bacteroidetes. In studies on the microbiome of the rhizosphere, members of the Proteobacteria and Firmicutes were described as the most dynamic taxa associated with disease suppression [20]. The potential implication of these bacterial taxa in protection of turtle eggs against *Fusarium* disease is not yet known.

No significant differences were detected in overall bacterial phyla composition between hatched and unhatched eggs or between the two nests. However, differences in abundance of two representative families of the microbial community of the sea turtle eggs were found. The significant difference in Pseudomonadaceae abundance among nests may reflect the variation in environmental conditions in the nesting area. Honarvar et al [23] demonstrated that bacterial diversity and richness increased with nest density and is higher in the zones closer to vegetation. *Pseudomonas* species have been previously isolated from cloaca of sea turtle females and eggs [22], [28]. They have been associated with diseases of captive sea turtles although their pathogenicity was not resolved [29]. In soil, the Pseudomonadaceae contribute to natural suppressiveness against several fungal pathogens including *Fusarium* [20], [30], [31]. For the Flavobacteriaceae, *C. indologenes* and *C. gleum* were the most represented OTUs (Welch test, p<0.01) in our PhyloChip analysis (Table S2). *Chryseobacterium indologenes* has been previously isolated from unhatched eggs of the loggerhead sea turtle *Caretta caretta* [22] and associated with shell disease of captive freshwater turtles [32]. Conversely, *Chryseobacterium* sp. strains are also known to exhibit antifungal activity [33]. Hence, the role of Flavobacteriaceae and/or the Pseudomonadaceae in mitigation of *Fusarium* infections of sea turtle eggs remains unclear. With the combined sample size of 4 eggshells, a first representative analysis of the microbial families that are associated with turtle eggs was performed (Figure 2). However, the differences observed between conditions (nests, hatched, unhatched) on family composition should be interpreted carefully due to the limited sample size per condition (n = 2).

The second most abundant bacterial phylum detected on the sea turtle eggshells was the Actinobacteria. Given their well-documented ability to produce an array of antibacterial and antifungal compounds [34]–[36], we isolated Actinobacteria from the eggshells and determined their activity against *F. falciforme*. The *in vitro* activity assays showed that isolated Actinobacteria of the genera *Streptomyces*, *Amycolaptosis*, *Micromonospora* and *Plantactinospora* are able to inhibit hyphal growth of *F. falciforme*. Interestingly, most of the antagonistic isolates described in this study were obtained from hatched eggs (Table 2). The majority of the antagonistic isolates belonged to the genus *Streptomyces* and this genus was the most representative group of the Actinobacteria (Table 2). In plants, *Streptomyces* species have been implicated in the protection against bacterial [37] and fungal pathogens including *Fusarium* [38], [39]. Species of the genus *Streptomyces* and other Actinobacteria with antifungal activity are also well known for their symbiotic associations with insects, protecting these from fungal pathogens [40]. The results of this study suggest that *Streptomyces* are a component of the bacterial community that reduce infection or proliferation of *Fusarium* on sea turtle eggs. Whether the *Streptomyces*, and other antagonistic Actinobacteria species, identified in this study can be used as a bioindicator, or as a component of protective microbiota in the nesting areas, to minimize sea turtle infections by *Fusarium* or other fungal pathogens remains to be investigated.

This study provides a first survey of the composition of the bacterial microflora on eggs of endangered sea turtles. Understanding not only the diversity and abundance of bacteria and other microorganisms associated with endangered species, but also the role of these microorganisms in disease suppression may have direct applications for nature conservation programs.

## Material and Methods

### Ethics Statement

Collection of sea turtle eggshells was done under permissions: 002 RM-DPM-MA and CITES 003/VS. None of the experiments involved sacrificing animals and, therefore, we did not require a specific approval from any institutional animal research ethics committee.

### Sample collection

Samples were collected from two selected nests of the sea turtle species *Eretmochelys imbricata* located in La Playita beach at Machalilla National Park (Ecuador) during the nesting season of 2012 (Figure S1). Four eggs (two hatched and two unhatched) were collected (Figure 1). Immediately after hatching of the eggs (approximately 45 days after the start of the incubation), one hatched and one unhatched egg (containing a nonviable embryo) were collected per nest, all with signs of *Fusarium* infection [41] (Figure 1). Samples were collected using sterile latex exam gloves and maintained at 4°C in individual bags during 2 days.

### Fungal isolation and molecular characterization

To confirm that the turtle eggs were indeed infected by *Fusarium* species, fragments of the eggshells (1 cm^2^) were placed on Peptone Dextrose Agar (PDA) and on Malt Agar (Figure S1), both supplemented with rifampicin (100 µg/ml) to prevent bacterial growth, and incubated at 25°C. Pure cultures of the fungal outgrowths were obtained by transferring single hyphal tips to fresh agar media. Pure cultures of the isolates are kept in the culture collection of the Laboratory of Phytopathology at Wageningen University, The Netherlands and the Real Jardín Botánico-CSIC, Spain.

To characterize the fungal isolates, DNA was extracted from mycelium (10 mg) collected from pure cultures. The mycelium was collected in 1.5 ml sterile tubes and 90 µl of NaOH (0.5 M) and two glass beads were added to the suspensions. The suspensions were placed in the Mixer Mill MM400 for 3 min to a frequency of 30 times/s, incubated at room temperature during 2 h and centrifuged at 13,000 rpm for 30 s. The supernatants were diluted 2 and 10 times with 0.1 M Tris-HCl (pH 7.0) for amplification. The primer pairs ITS1/ITS4 were used to amplify the internal transcribed spacer of the nuclear ribosomal DNA (ITS nrDNA). Amplification reactions were performed in 50 µl of reaction that contained 4 µl of DNA sample, 10 µl of 5x Colorless GoTaq Reaction buffer (Promega Co. Ma, US), 2 µl of each primer (10 µM), 2 µl of mix of dNTPs (5 mM), 0.2 µl of 5 U/µl GoTaq DNA polymerase (Promega Co. Ma, US) and 29.8 µl of MiliQ water. The amplification program was: initial denaturalization at 94°C for 5 min; 35 cycles of 94°C for 1 min, 60°C for 1 min and 72°C for 2 min; with a final extension at 72°C for 5 min.

The amplification products were sequenced in both forward and reverse direction (MACROGEN, Amsterdam, The Netherlands). Sequencing results were processed by Sequencher 4.2 (Gene Codes Corporation, Ann Arbor, Michigan, USA) and initially compared with sequences in the National Centre of Biotechnology Information (NCBI) nucleotide databases using BLAST [42]. For precise identification of the *Fusarium* spp. a phylogenetic analyses was carried out. The generated ITS nrDNA sequences from isolated *Fusarium* (Table 1), 136 NCBI-GenBank sequences of *Fusarium* turtle egg isolates (Table S1), and 60 selected sequences of *Fusarium* spp. from other hosts and environments were included (Table S2). The program Se-Al 2.0a11 Carbon [43] was used for manual alignment of the sequences. Maximum parsimony analysis (MP) [44] was inferred using the heuristic search option in PAUP*v4.0b10. Nonparametric bootstrap support (BS) [45] for each clade was tested based on 10,000 replicates, using the fast-step option. Newly obtained sequences were submitted to GenBank with accession numbers KF179246 through KF179255.

### Bacterial isolation and DNA extraction from sea turtle eggshells

For bacterial isolation and DNA extraction, the eggshells (4 cm^2^) were individually suspended in 10 ml of sterile tap water and vortexed for 2 min. The suspensions were sonicated using an ultrasonic bath (Transsonic 460, Elma) for 2 min and vortexed for an additional 2 min at maximum speed. Each suspension was divided in 1.5 ml aliquots in eppendorf tubes and centrifuged at 13,000 rpm for 30 min. Pellets were resuspended in 100 µl of sterile tap water by vortexing and pipetting and then pooled in a sterile eppendorf tube to a final volume of approximately 700 µl. A 50 µl aliquot of each suspension was mixed with 50 µl of 80% glycerol and these samples were stored in the freezer at −20°C until processed for bacterial isolations. The remaining suspension (approximately 650 µl) was centrifuged at 13,000 rpm during 30 min, supernatants were discarded and pellets were stored at −80°C until processed for DNA extraction. For bacterial isolations, glycerol suspensions were diluted in 10-fold steps up to 10,000 times and, for each dilution, two replicates of 50 µl were plated on 1/10th TSA for total aerobic bacteria and on the semi-selective medium GA supplemented with Nalidixic acid (20 µg/ml) and Trimethoprim (20 µg/ml) for Actinobacteria (Figure S1). Both media were additionally supplemented with Delvocid (100 µg/ml) to prevent fungal growth. TSA plates were incubated at 25°C for 5 days and GA plates were incubated at 30°C for 21 days. Colonies were collected from GA medium. Based on the colony counts, the number of CFU/cm^2^ was calculated.

### PhyloChip analysis

To identify the bacterial and archaeal communities on the shells of *Fusarium*–infected sea turtle eggs, metagenomic DNA was isolated from the cell pellets extracted from the hatched and unhatched eggs (Figure S1). The PowerSoil DNA Isolation Kit (MO BIO Laboratories, Inc.) was used for DNA isolation according to the manufacturer's instructions. The DNA concentration was determined by a Nanodrop 1000 Spectrophotometer (Thermo Scientific). The microbial profile for each sample was generated by G3-PhyloChip analysis (Second Genome, CS, USA). All PCR conditions and universal primers used for amplification of 16S rDNA genes of bacteria and archaea were previously described by [18]. Fragmentation of the 16S rDNA amplicons, labelling, hybridization, staining, and scanning of the PhyloChip, as well as data processing to determine absence/presence and HybScores of OTUs was performed according to methods described by [18]. Phyla represented by over 10% of the detected OTUs were analysed in detail. These Phyla were also analysed at the family and genus level. Comparisons of composition between samples were performed using the Bray-Curtis distance with the average as the clustering method. Statistical analyses of the PhyloChip data were performed by Primer-E 6 software (PRIMER-E *Ltd*., UK).

### *In vitro* activity assay and BOX-PCR based identifications of Actinobacteria

Because Actinobacteria have the ability to produce an array of antibacterial and antifungal compounds [34]–[36], all the bacterial isolates obtained from GA medium were purified and screened for *in vitro* antagonism against *Fusarium* isolate 331FUS, which was obtained from the sea turtle eggs in this study. For each bacterial isolate, one 5 mm diameter agar plug from 3-week-old culture plates was inoculated at the periphery of a quadrant of 1/5 strength PDA plates (four plugs per plate in total) and incubated for 4 days at 30°C. After this period, a 5 mm diameter agar plug from a 7-day-old *Fusarium* plate culture was transferred to the centre of the plate. After an additional 7 days of incubation at 30°C, inhibition of hyphal growth by each of the four bacterial isolates was measured and expressed relative to radial hyphal growth of *Fusarium* on plates without bacteria.

The genotypic diversity of the bacterial isolates with antagonist activity against *Fusarium* was assessed by BOX-PCR using the 22-mer BOXA1R oligonucleotide [46], [47]. DNA was extracted from pure cultures using 2 mg of the colonies by microwave treatment as described previously [48]. The suspensions were centrifuged at 13,000 rpm for 30 s and supernatants were used for amplifications. Amplification reactions were performed in 25 µl containing 1 µl of DNA sample, 5 µl of 5x Gitschier buffer [49], 1 µl the BOX1AR primer (10 µm), 1.25 µl of mix of dNTPs (100 mM), 0.4 µl of BSA (10 mg/ml), 2.5 µl of 100% DMSO, 0.4 µl of 5 U/µl GoTaq DNA polymerase (Promega Co. Ma, US) and 13.45 µl of MiliQ water. Amplification was performed following an initial denaturation at 95°C for 2 min; 30 cycles at 94°C for 3 s, 92°C for 30 s, 50°C for 1 min and 65°C for 8 min, with a final extension at 65°C for 8 min [50]. PCR amplification products were detected by electrophoresis in 1% (w/v) agarose gels (5h at 45W). DNA fingerprints were visually compared for similarity; variations in intensity of bands were not taken into account in the analysis. For one isolate of each specific BOX group, the 16S rDNA was amplified with primer pair 8F/1392R [51]. The amplification products were sequenced both forward and reverse (MACROGEN, Amsterdam, The Netherlands). Sequences were processed by Sequencher 4.2 (Gene Codes Corporation, Ann Arbor, Michigan, USA) to obtain the sequence for each isolate. Sequences obtained were compared with those in the NCBI and GreenGenes databases (greengenes.lbl.gov/cgi-bin/nph-blast_interface.cgi). The sequences have been submitted to GenBank with accession numbers KF179216 through KF179245.

The Phylogenetic relationship of the isolated antagonistic Actinobacteria and the Actinobacteria OTUs detected by the PhyloChip was determined per genera. Additional GenBank sequences of *Solwarapora* sp. (JN633950, JN633958 and JN633962) and *Platactinospora* sp. (KC336252 and FJ214343) were included in the analysis. The 16S rDNA sequences of *Streptomyces ambifaciens* (M27245) and *Solwaraspora* sp. (JN633950) were included as outgroups in the analysis of non-corresponding genera, respectively. The tool MUSCLE available in MEGA5.05 [52] was used to align the 16S rDNA sequences. Maximum parsimony analyses (MP) and BS support where inferred following the methodology explained above.

## Supporting Information

Figure S1 **Schematic presentation of the metagenomic and classical microbiological approaches and techniques.** The scheme represent the approaches used to isolate, identify and characterize the fungal and bacterial community from eggs of the sea turtle species *Eretmochelys imbricata* nesting at La Playita beach, Machalilla National Park, Ecuador.
(TIF)

Figure S2 **Out-group rooted cladogram of the ITS nrDNA region of isolates within the** ***Fusarium solani*** **species complex.** One of the most parsimonious trees inferred from the ITS nrDNA sequence data of 136 sea turtle fungal isolates and 60 non-sea turtle fungal isolates. The numbers on the internodes indicate the bootstrap values (BS) of the parsimony analysis. Highlighted isolates correspond to those obtained in this work (*n* = 10). The arrow indicates the *F. falciforme* isolate, *i.e*., 331FUS, used in the dual culture assays to determine the activity of the Actinobacteria.
(TIF)

Figure S3 **Cluster analysis (Bray-Curtis) of the microbiome of hatched and unhatched eggs infected by** ***Fusarium falciforme*** **.** A) Dendogram of family Pseudomonadaceae (*n* = 949 OTUs). B) Dendogram of family Flavobacteriaceae (*n* = 710 OTUs). Abbreviations as in Figure 2.
(TIF)

Figure S4 **Out-group rooted phylogenetic tree inferred from the 16S rDNA sequence data from isolates of** ***Streptomyces*** **spp.** Data includes isolates of ***Streptomyces*** spp. (*n* = 16) with activity against *Fusarium falciforme*, and those detected by the PhyloChip analysis (*n* = 364). The numbers at the internodes indicate the bootstrap values (BS) of the parsimony analysis.
(TIF)

Figure S5 **Out-group rooted phylogenetic trees inferred from sequence data from isolates of the** ***Amycolaptosis*** **sp. and Micromonosporaceae.** Phylogenetic trees were inferred from the 16S rDNA data from isolates from both taxa, with activity against *Fusarium falciforme*, and those detected by the PhyloChip analysis. A) Phylogenetic tree from the isolates of the *Amycolaptosis sp.* (*n* = 3) with activity against *F. falciforme*, and those detected by the PhyloChip analysis (*n* = 29). B) Phylogenetic tree from isolates of the Micromonosporaceae (*n* = 11) with activity against *F. falciforme*, those detected by the PhyloChip analysis (*n* = 33), and additional GenBank strains (*n* = 5). The numbers at the internodes of the phylogenetic trees indicate the bootstrap values (BS) of the parsimony analysis.
(TIF)

Table S1 **Number of bacteria isolated from the shells of hatched and unhatched eggs of the sea turtle species** ***Eretmochelys imbricata*** **on 1/10^th^ TSA agar medium (total aerobic bacteria) and on GA medium (semi-selective for Actinobacteria).** Presented are the Colony Forming Units (CFU/cm^2^) for each of the two media and for each of the two hatch statuses. For each hatch status, a mean value of 2 eggs is given. SD refers to the standard deviation.
(DOCX)

Table S2 **Most abundant microbial communities from** ***Fusarium*** **-infected eggshells of the sea turtle species** ***Eretmochelys imbricata*** **.** Data shown represent the most abundant phyla and families detected by the PhyloChip. The families highlighted in grey are most represented (with >10%) per phylum.
(DOCX)

Table S3 ***Chryseobacterium*** **species found significantly more abundant on eggshells of hatched than of unhatched eggs of the sea turtle species** ***Eretmochelys imbricata*** **(Welsh test, p<0.01;** ***r*** **= 1, Anosim).**
(DOCX)
