# Supplementary figures and images for: Unravelling the Microbiome of Eggs of the Endangered Sea Turtle Eretmochelys imbricata Identifies Bacteria with Activity against the Emerging Pathogen Fusarium falciforme
Source: PLoS One. 2014 Apr 17;9(4):e95206. doi: 10.1371/journal.pone.0095206 (PMC3990731; doi:10.1371/journal.pone.0095206)

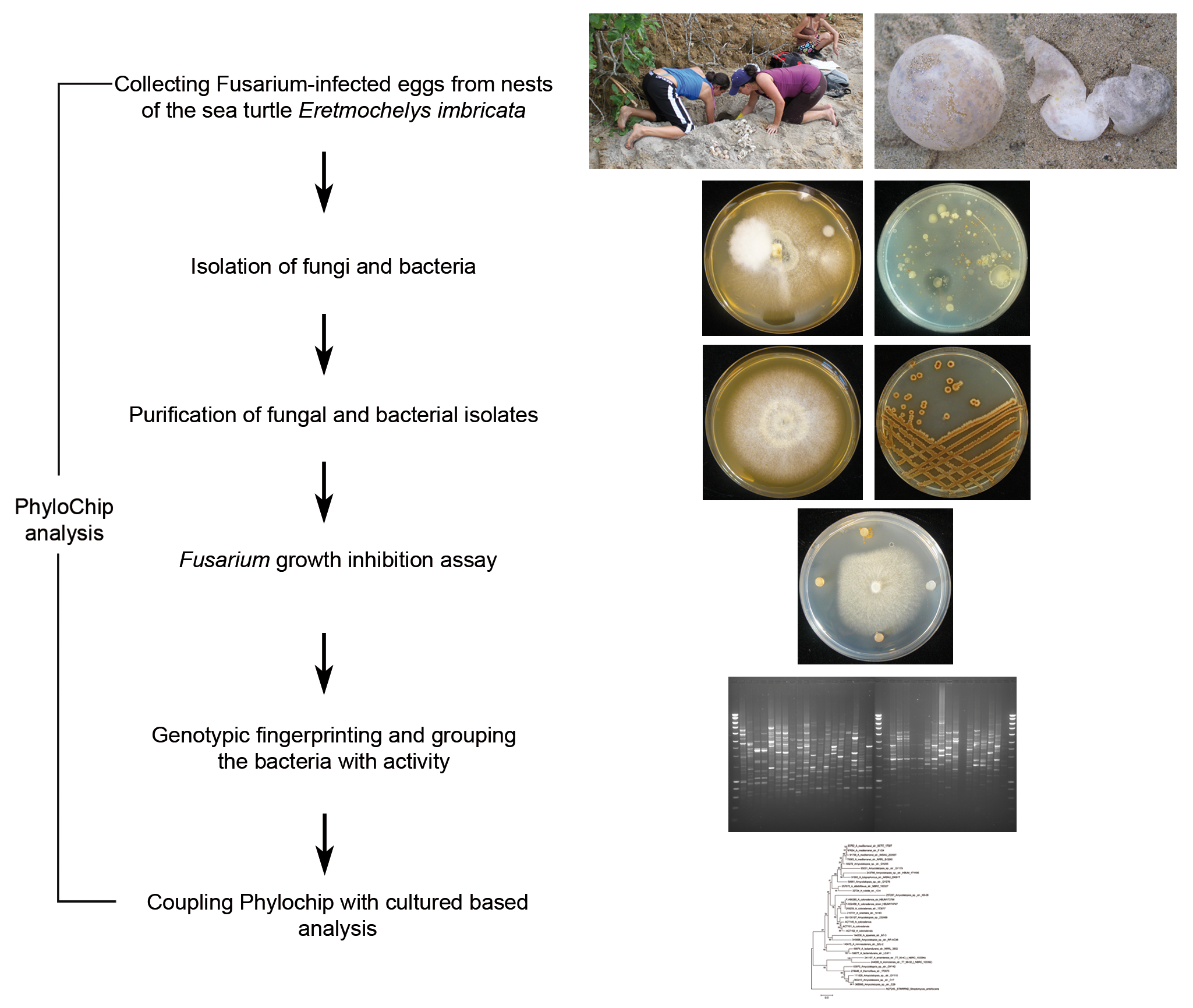

Supplement: Figure S1 — Schematic presentation of the metagenomic and classical microbiological approaches and techniques. The scheme represent the approaches used to isolate, identify and characterize the fungal and bacterial community from eggs of the sea turtle species Eretmochelys imbricata nesting at La Playita beach, Machalilla National Park, Ecuador. (TIF) [file pone.0095206.s001.tif]

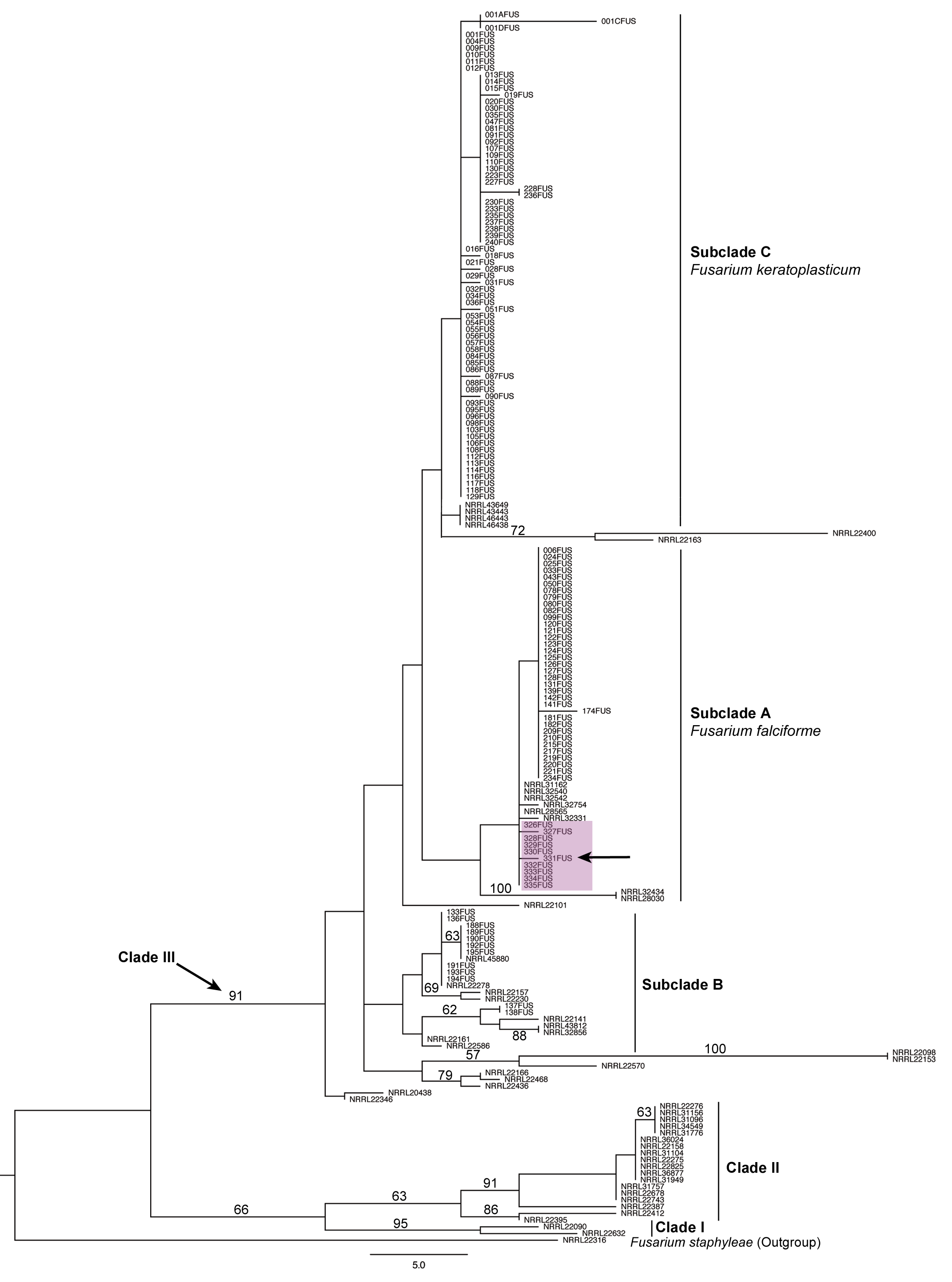

Supplement: Figure S2 — Out-group rooted cladogram of the ITS nrDNA region of isolates within the Fusarium solani species complex. One of the most parsimonious trees inferred from the ITS nrDNA sequence data of 136 sea turtle fungal isolates and 60 non-sea turtle fungal isolates. The numbers on the internodes indicate the bootstrap values (BS) of the parsimony analysis. Highlighted isolates correspond to those obtained in this work (n = 10). The arrow indicates the F. falciforme isolate, i.e., 331FUS, used in the dual culture assays to determine the activity of the Actinobacteria. (TIF) [file pone.0095206.s002.tif]

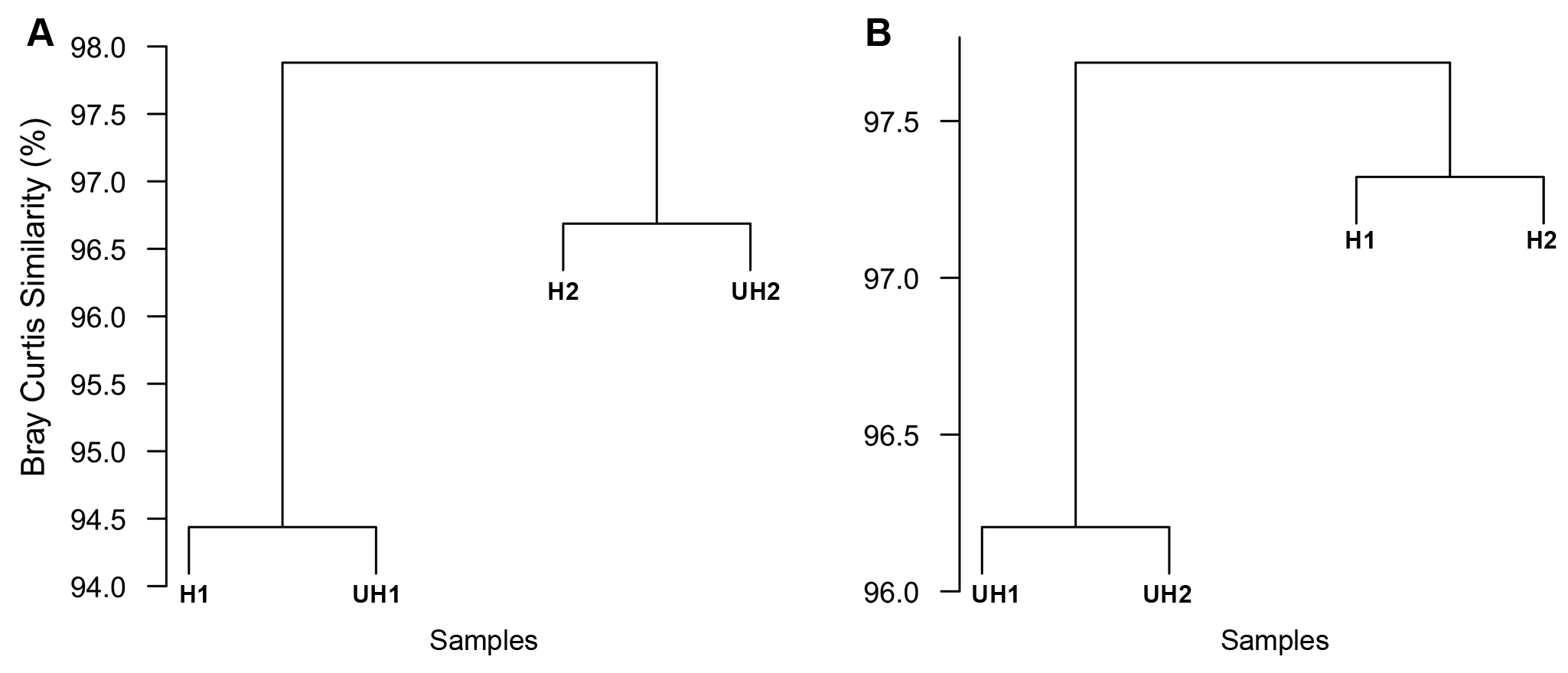

Supplement: Figure S3 — Cluster analysis (Bray-Curtis) of the microbiome of hatched and unhatched eggs infected by Fusarium falciforme . A) Dendogram of family Pseudomonadaceae (n = 949 OTUs). B) Dendogram of family Flavobacteriaceae (n = 710 OTUs). Abbreviations as in Figure 2. (TIF) [file pone.0095206.s003.tif]

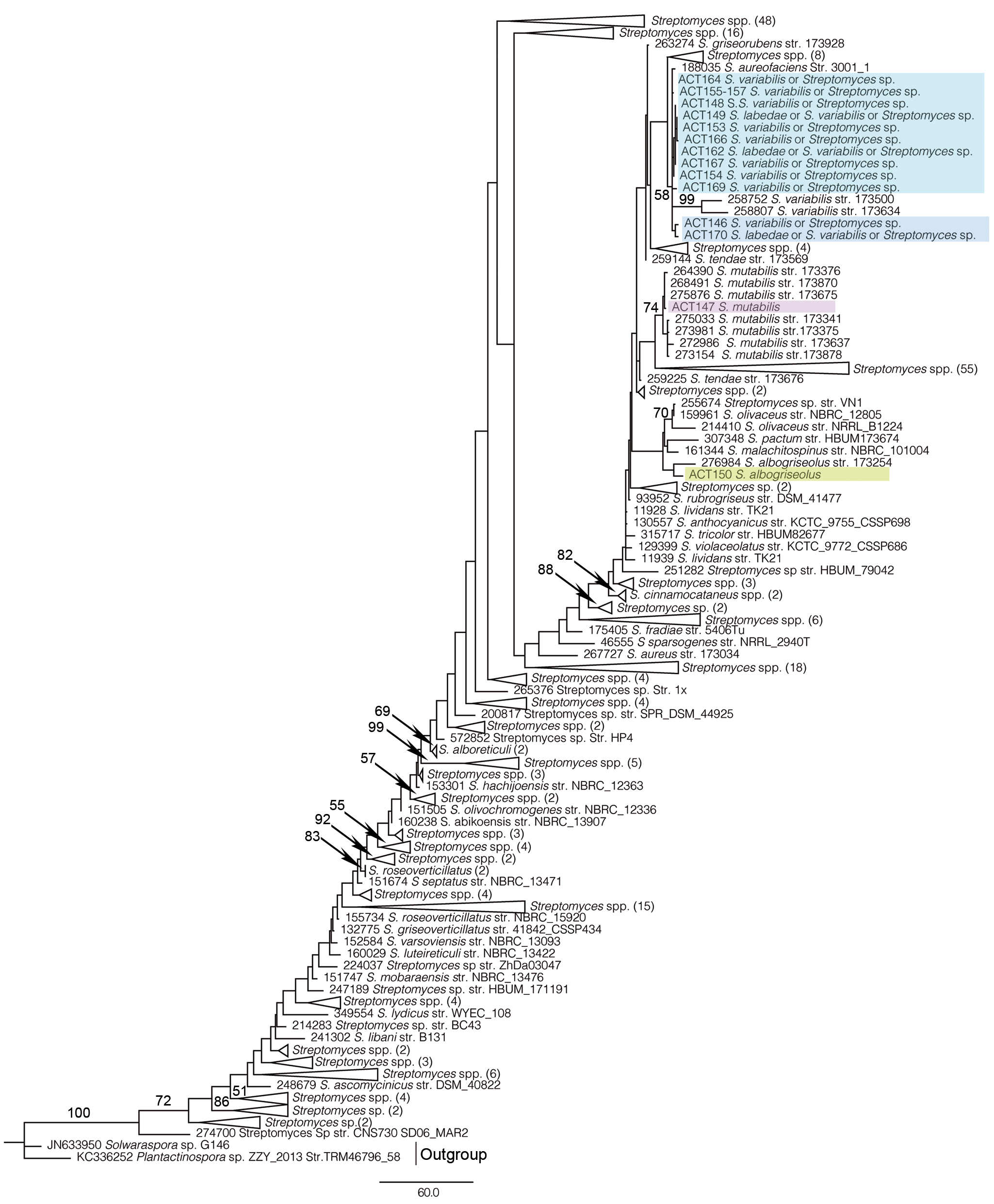

Supplement: Figure S4 — Out-group rooted phylogenetic tree inferred from the 16S rDNA sequence data from isolates of Streptomyces spp. Data includes isolates of Streptomyces spp. (n = 16) with activity against Fusarium falciforme, and those detected by the PhyloChip analysis (n = 364). The numbers at the internodes indicate the bootstrap values (BS) of the parsimony analysis. (TIF) [file pone.0095206.s004.tif]

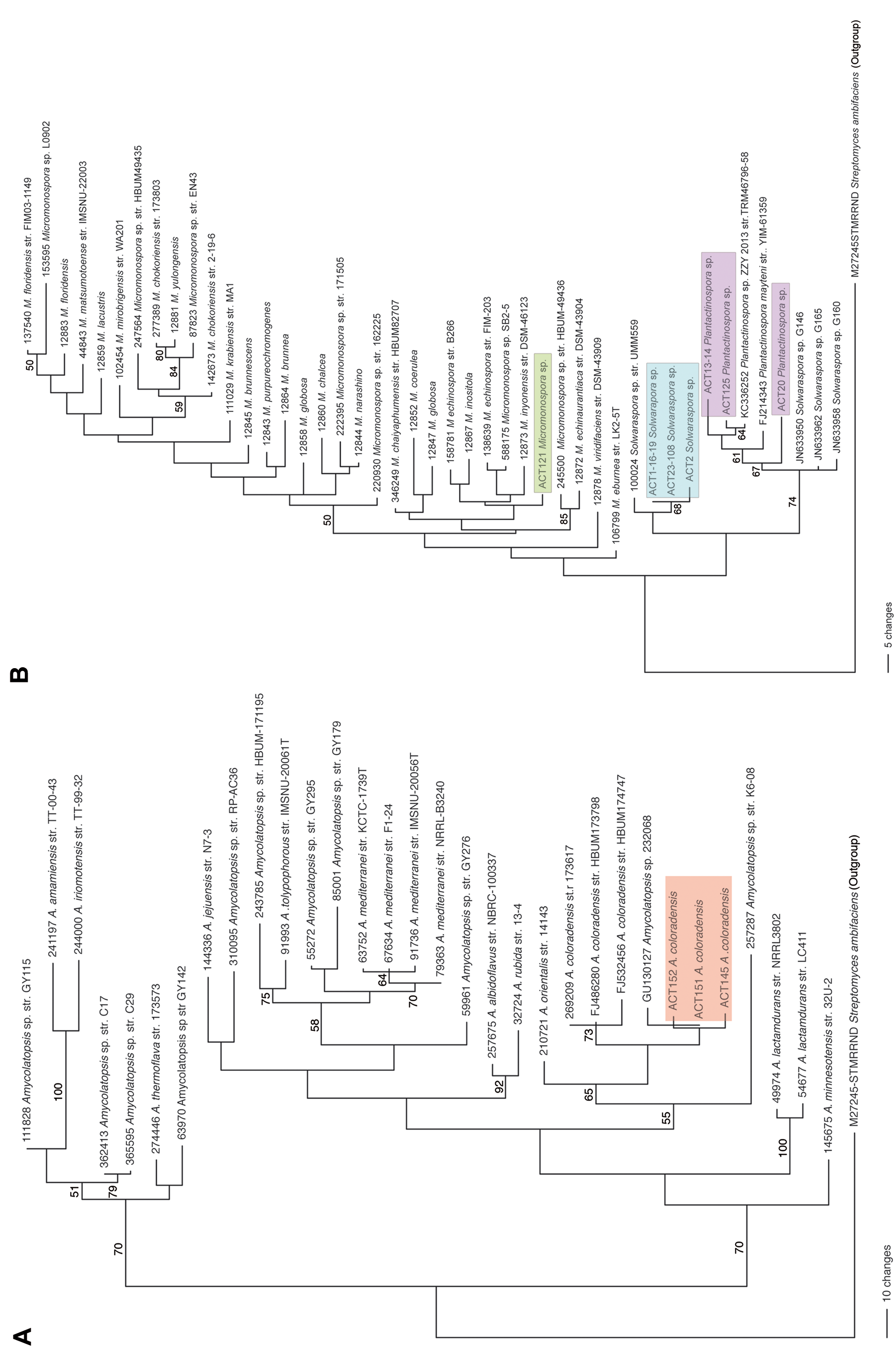

Supplement: Figure S5 — Out-group rooted phylogenetic trees inferred from sequence data from isolates of the Amycolaptosis sp. and Micromonosporaceae. Phylogenetic trees were inferred from the 16S rDNA data from isolates from both taxa, with activity against Fusarium falciforme, and those detected by the PhyloChip analysis. A) Phylogenetic tree from the isolates of the Amycolaptosis sp. (n = 3) with activity against F. falciforme, and those detected by the PhyloChip analysis (n = 29). B) Phylogenetic tree from isolates of the Micromonosporaceae (n = 11) with activity against F. falciforme, those detected by the PhyloChip analysis (n = 33), and additional GenBank strains (n = 5). The numbers at the internodes of the phylogenetic trees indicate the bootstrap values (BS) of the parsimony analysis. (TIF) [file pone.0095206.s005.tif]
