# Supplementary material for: Unravelling the Microbiome of Eggs of the Endangered Sea Turtle Eretmochelys imbricata Identifies Bacteria with Activity against the Emerging Pathogen Fusarium falciforme
Source: PLoS One. 2014 Apr 17;9(4):e95206. doi: 10.1371/journal.pone.0095206 (PMC3990731; doi:10.1371/journal.pone.0095206)
Supplement: Table S1 — Number of bacteria isolated from the shells of hatched and unhatched eggs of the sea turtle species Eretmochelys imbricata on 1/10th TSA agar medium (total aerobic bacteria) and on GA medium (semi-selective for Actinobacteria). Presented are the Colony Forming Units (CFU/cm2) for each of the two media and for each of the two hatch statuses. For each hatch status, a mean value of 2 eggs is given. SD refers to the standard deviation. (DOCX) [file pone.0095206.s006.docx]

**Table S1.** Number of bacteria isolated from the shells of hatched and unhatched eggs of the sea turtle species *Eretmochelys imbricata* on 1/10^th^ TSA agar medium (total aerobic bacteria) and on GA medium (semi-selective for Actinobacteria). Presented are the Colony Forming Units (CFU/cm^2^) for each of the two media and for each of the two hatch statuses. For each hatch status, a mean value of 2 eggs is given. SD refers to the standard deviation.

| Media | Sample | Percentage |
| --- | --- | --- |
| 1/10^th^ TSA | Hatched | 3.1x10^7^(+1.9x10^7^) |
|  | Unhatched | 8.7x10^7^ (+3.1x10^7^) |
| GA | Hatched | 1.2x10^4^(+1.6x10^3^) |
|  | Unhatched | 2x10^5^(+ 2.7x10^5^) |
