# Supplementary material for: Unravelling the Microbiome of Eggs of the Endangered Sea Turtle Eretmochelys imbricata Identifies Bacteria with Activity against the Emerging Pathogen Fusarium falciforme
Source: PLoS One. 2014 Apr 17;9(4):e95206. doi: 10.1371/journal.pone.0095206 (PMC3990731; doi:10.1371/journal.pone.0095206)
Supplement: Table S2 — Most abundant microbial communities from Fusarium -infected eggshells of the sea turtle species Eretmochelys imbricata . Data shown represent the most abundant phyla and families detected by the PhyloChip. The families highlighted in grey are most represented (with >10%) per phylum. (DOCX) [file pone.0095206.s007.docx]

**Table S2.** Most abundant microbial communities from *Fusarium*-infected eggshells of the sea turtle species *Eretmochelys imbricata.* Data shown represent the most abundant phyla and families detected by the PhyloChip. The families highlighted in grey are most represented (with >10%) per phylum.

| Phylum | Class | Family | OTUs | Percentage^*^ |
| --- | --- | --- | --- | --- |
| Proteobacteria^*^ | Gamma- | *Enterobacteriaceae* | 1154 | 29.41 |
|  |  | *Pseudomonadaceae* | 949 | 24.18 |
|  |  | *Xanthomonadaceae* | 295 | 7.52 |
|  |  | *Moraxellaceae* | 219 | 5.58 |
|  |  | *Chromatiaceae* | 137 | 3.49 |
|  |  | *Halomonadaceae* | 128 | 3.26 |
|  |  | *Alteromonadaceae* | 115 | 2.93 |
|  |  | *Vibrionaceae* | 100 | 2.55 |
|  |  | *Sinobacteraceae* | 64 | 1.63 |
|  |  | *unclassified* | 61 | 1.55 |
|  |  | Others |  | 17.89 |
|  | Alpha- | *Rhodobacteriaceae* | 404 | 18.14 |
|  |  | *Rhizobiaceae* | 268 | 12.03 |
|  |  | *Sphingomonadaceae* | 231 | 10.37 |
|  |  | *Rhodospirillaceae* | 219 | 9.83 |
|  |  | *Phyllobacteriaceae* | 182 | 5.93 |
|  |  | *Bradyrhizobiaceae* | 132 | 5.93 |
|  |  | *Hyphomicrobiacea* | 122 | 5.48 |
|  |  | *Unclassified* | 109 | 4.89 |
|  |  | *Caulobacteriaceae* | 103 | 4.63 |
|  |  | *Erythrobacteriaceae* | 78 | 3.50 |
|  |  | *Pelagibacteriaceae* | 69 | 3.10 |
|  |  | Others |  | 16.17 |
|  | Beta- | *Comamonadaceae* | 889 | 46.13 |
|  |  | *Aquabacteriaceae* | 294 | 15.26 |
|  |  | *Burkholderiaceae* | 181 | 9.39 |
|  |  | *Alcaligenaceae* | 180 | 9.34 |
|  |  | *Oxalobacteraceae* | 81 | 4.20 |
|  |  | *Neisseriaceae* | 71 | 3.68 |
|  |  | *Rhodocyclaceae* | 68 | 3.53 |
|  |  | *unclassified* | 59 | 3.06 |
|  |  | Others |  | 5.40 |
|  | Delta- | *Unclassified* | 106 | 26.63 |
|  |  | *Myxococcaceae* | 42 | 10.55 |
|  |  | *Desulfobacteriaceae* | 37 | 9.30 |
|  |  | *Syntrophobacteriaceae* | 29 | 7.29 |
|  |  | *Nitrospinaceae* | 23 | 5.78 |
|  |  | *Haliangiaceae* | 20 | 5.03 |
|  |  | Others |  | 35.43 |
| Actinobacteria |  | *Corynebacteriaceae* | 561 | 19.38 |
|  |  | *Streptomycetaceae* | 453 | 15.65 |
|  |  | *Microbacteriaceae* | 345 | 11.92 |
|  |  | *Micrococcaceae* | 218 | 7.53 |
|  |  | *Mycobacteriaceae* | 130 | 4.49 |
|  |  | *Nocardiaceae* | 114 | 3.94 |
|  |  | *Micromonosporaceae* | 109 | 3.77 |
|  |  | *Nocardioidaceae* | 96 | 3.32 |
|  |  | *unclassifed* | 78 | 2.70 |
|  |  | *Pseudonocardiaceae* | 77 | 2.66 |
|  |  | *Cellulomonadaceae* | 63 | 2.18 |
|  |  | *Propionibacteriaceae* | 64 | 2.21 |
|  |  | *Thermomonosporaceae* | 52 | 1.80 |
|  |  | *Intrasporangiaceae* | 41 | 1.42 |
|  |  | *Coriobacteriaceae* | 41 | 1.42 |
|  |  | *Streptosporangiaceae* | 39 | 1.35 |
|  |  | *Nocardiopsaceae* | 34 | 1.17 |
|  |  | Others |  | 13.10 |
| Firmicutes |  | *Lachnospiraceae* | 559 | 22.62 |
|  |  | *Bacillaceae* | 526 | 21.29 |
|  |  | *Ruminococcaceae* | 371 | 15.01 |
|  |  | *Paenibacillaceae* | 125 | 5.06 |
|  |  | *Planococcaceae* | 118 | 4.78 |
|  |  | *Staphylococcaceae* | 111 | 4.49 |
|  |  | *Streptoccaceae* | 106 | 4.29 |
|  |  | *Clostridiaceae* | 77 | 3.12 |
|  |  | *Lactobacillaceae* | 71 | 2.87 |
|  |  | *Veillonellaceae* | 53 | 2.14 |
|  |  | *Enterococcaceae* | 46 | 1.86 |
|  |  | *Unclassified* | 38 | 1.54 |
|  |  | *Carnobacteriaceae* | 36 | 1.46 |
|  |  | Others |  | 9.47 |
| Bacteroidetes |  | *Flavobacteriaceae* | 710 | 51.26 |
|  |  | *RikenellaceaeII* | 153 | 11.05 |
|  |  | *Chitinophagaceae* | 87 | 6.28 |
|  |  | *Sphingobacteriaceae* | 80 | 5.78 |
|  |  | *Prevotellaceae* | 60 | 4.33 |
|  |  | *Flexibacteraceae* | 46 | 3.32 |
|  |  | *Porphyromonadaceae* | 34 | 2.45 |
|  |  | *Flammeovirgaceae* | 34 | 2.45 |
|  |  | *Rikenellaceae* | 33 | 2.38 |
|  |  | Others |  | 10.69 |

* Percentages stated for Proteobacteria where calculated per class. For the other families percentages were calculated per phylum.
