# Supplementary material for: Unravelling the Microbiome of Eggs of the Endangered Sea Turtle Eretmochelys imbricata Identifies Bacteria with Activity against the Emerging Pathogen Fusarium falciforme
Source: PLoS One. 2014 Apr 17;9(4):e95206. doi: 10.1371/journal.pone.0095206 (PMC3990731; doi:10.1371/journal.pone.0095206)
Supplement: Table S3 — Chryseobacterium species found significantly more abundant on eggshells of hatched than of unhatched eggs of the sea turtle species Eretmochelys imbricata (Welsh test, p<0.01; r = 1, Anosim). (DOCX) [file pone.0095206.s008.docx]

**Table S3.** *Chryseobacterium* species found significantly more abundant on eggshells of hatched than of unhatched eggs of the sea turtle species *Eretmochelys imbricata* (Welsh test, p<0.01; *r*= 1, Anosim).

| Species | OTUs | Percentage |
| --- | --- | --- |
| *C. indologenes* | 5 | 6.76 |
| *C. bovis* | 1 | 1.35 |
| *C. gleum* | 4 | 5.41 |
| *C. daecheongense* | 2 | 2.70 |
| *C. luteum* | 1 | 1.35 |
| Unclassified | 61 | 82.43 |

* Percentage that each species represented for the genus *Chryseobacterium*.
